# Development of a multi‐faceted intervention for hearing/vision support for residents with dementia in long term care'

**DOI:** 10.1002/alz.092595

**Published:** 2025-01-09

**Authors:** Iracema Leroi

**Affiliations:** ^1^ Global Brain Health Institute, Trinity College Dublin, Dublin, Ireland; School of Medicine and Global Brain Health Institute, Trinity College Dublin, Dublin Ireland

## Abstract

**Context:**

The development of new effective and sustainable dementia care interventions requires active engagement of all service users in research studies. This ensures that interventions are tailored to meet individual needs and preferences. Here, we describe modelling and development of a multi‐faceted sensory health support intervention for residents with dementia in long‐term care, co‐designed with dementia care users and their supporters. There is a high rate of unrecognised hearing and vision loss in people with dementia, particularly in long term care (LTC) settings, which can hasten cognitive and functional decline, contribute to neuropsychiatric challenges, and increase the impact on health resource utilization.

**Methods:**

We undertook a multistage process with key user stakeholders, interdisciplinary health experts, LTC professionals, and Patient and Public Involvement (PPI) representatives, following the UK’s Medical Research Council’s framework for the development of complex interventions.

In the first stage, we crafted an outline of the intervention by extending a previously evaluated home‐based sensory support intervention and incorporating evidence from a systematic survey of hearing rehabilitation interventions. The ‘Sensory‐Cognitive Model of Place’ served as the theoretical framework.

In the second stage, qualitative feedback from professional and PPI stakeholders, along with insights into existing care sensory health training approaches, were integrated to form a multi‐component sensory support intervention ready for field testing.

The third stage involved pilot‐feasibility trialing in a single LTC facility, with subsequent design modifications based on feedback.

The resulting *Sensory Support Intervention for Residents with Dementia in Care* (SSI‐RC) comprises four tiered components addressing (1) resident, (2) staff, (3) environmental, and (4) organizational levels. These include personalized sensory health status checks, staff awareness training, sensory environment audits, and organizational pathway assessments.

**Conclusion:**

With the guidance of service users, we successfully developed and field tested the SSI‐RC, highlighting its potential for implementation in a cluster‐randomized pilot trial in LTC facilities. The collaborative approach with dementia care users and supporters ensures the intervention’s relevance and effectiveness in enhancing sensory‐cognitive health for residents with dementia. The research contributes to the broader goal of tailoring dementia care interventions to individual needs and preferences.

